# Recent Advances in Micro- and Nanorobot-Assisted Colorimetric and Fluorescence Platforms for Biosensing Applications

**DOI:** 10.3390/mi15121454

**Published:** 2024-11-29

**Authors:** Arumugam Selva Sharma, Nae Yoon Lee

**Affiliations:** 1 Department of Nanoscience and Technology, Gachon University, 1342 Seongnam-daero, Sujeong-gu, Seongnam-si 13120, Gyeonggi-do, Republic of Korea; 2 Department of BioNano Technology, Gachon University, 1342 Seongnam-daero, Sujeong-gu, Seongnam-si 13120, Gyeonggi-do, Republic of Korea

**Keywords:** micro- and nanorobots, actuation, colorimetry, fluorescence, biosensing, diagnostics

## Abstract

Micro- and nanorobots (MNRs) have attracted significant interest owing to their promising applications in various fields, including environmental monitoring, biomedicine, and microengineering. This review explores advances in the synthetic routes used for the preparation of MNRs, focusing on both top-down and bottom-up approaches. Although the top-down approach dominates the field because of its versatility in design and functionality, bottom-up strategies that utilize template-assisted electrochemical deposition and bioconjugation present unique advantages in terms of biocompatibility. This review investigates the diverse propulsion mechanisms employed in MNRs, including magnetic, electric, light, and biological forces, which enable efficient navigation in various fluidic environments. The interplay between the synthesis and propulsion mechanisms of MNRs in the development of colorimetric and fluorescence detection platforms is emphasized. Additionally, we summarize the recent advancements in MNRs as sensing and biosensing platforms, particularly focusing on colorimetric and fluorescence-based detection systems. By utilizing the controlled motion of MNRs, dynamic changes in the fluorescent signals and colorimetric responses can be achieved, thereby enhancing the sensitivity and selectivity of biomolecular detection. This review highlights the transformative potential of MNRs in sensing applications and emphasizes their role in advancing diagnostic technologies through innovative motion-driven signal transduction mechanisms. Subsequently, we provide an overview of the primary challenges currently faced in MNR research, along with our perspective on the future applications of MNR-assisted colorimetric and fluorescence biosensing in chemical and biological sensing. Moreover, issues related to enhanced stability, biocompatibility, and integration with existing detection systems are discussed.

## 1. Introduction

The field of micro/nanorobots (MNRs) has its conceptual roots in the visionary ideas of Richard Feynman, who, in his 1959 speech “There’s Plenty of Room at the Bottom”, proposed the use of small machines to treat diseases within the human body [1,2,3]. By the 1990s, the theoretical foundation established by Feynman began to materialize with significant advances in molecular machines. This progress has been marked by the cutting-edge works of Jean-Pierre Sauvage, Fraser Stoddart, and Ben Feringa [4]. Their pioneering efforts in the design and synthesis of these molecular systems led to the 2016 Nobel Prize in Chemistry. In 1999, Feringa et al. achieved a significant breakthrough by developing the first rotary molecular motor [5]. This study successfully addressed the longstanding challenge of creating continuous and unidirectional movement within molecular systems. Subsequently, significant advancements in molecular shuttles, nanocars, and molecular muscles have expanded the capabilities of these systems [6]. These studies have further facilitated the integration of molecular/nanomachines into various fields, including molecular electronics and catalysis, showcasing their versatility in performing mechanical work and triggering complex molecular operations [4]. Typically, MNRs are defined as micro- to nanoscale devices capable of converting various forms of energy into kinetic energy, thereby enabling autonomous movement. MNR devices are engineered to operate at dimensions typically below 1 mm, with microrobots categorized as those ranging from 1 µm to 1 mm in size, and nanorobots as those smaller than 1 µm [3,7,8,9]. MNRs are powered through a variety of mechanisms, either chemically, by utilizing locally accessible chemical fuels, or physically, through external fields such as magnetic, light, ultrasound, or electric fields [3,7,8,9]. In some cases, biomolecules, microorganisms, or cells have been used to control the MNR movement. In general, MNRs operating fluid environments are primarily influenced by inertial and viscous forces [10]. The relative significance of these forces is described by the Reynolds number, which provides a dimensionless ratio of the inertial to viscous forces in a fluid flow. For larger organisms such as fish, movement occurs at high Reynolds numbers, where inertial forces dominate and facilitate efficient propulsion [10,11]. In contrast, for microorganisms and, by extension, micro/nanorobots, movement occurs at very low Reynolds numbers [12,13]. In this context, viscous forces dominate, whereas inertial effects are negligible, suggesting that MNRs must continuously spend energy to sustain their motion. Consequently, overcoming the viscous resistance inherent in fluidic environments at the micro- and nanoscale poses a significant challenge. This necessitates continuous actuation to maintain movement because these small-scale robots must consistently counteract the drag forces exerted by the surrounding medium to achieve effective propulsion and operational efficiency. Owing to their ability to navigate and manipulate their environment independently, MNRs hold significant promise for applications in sensing, microsurgery, targeted drug delivery, and noninvasive or minimally invasive diagnostic and therapeutic procedures [10,11].

**Scheme 1:**
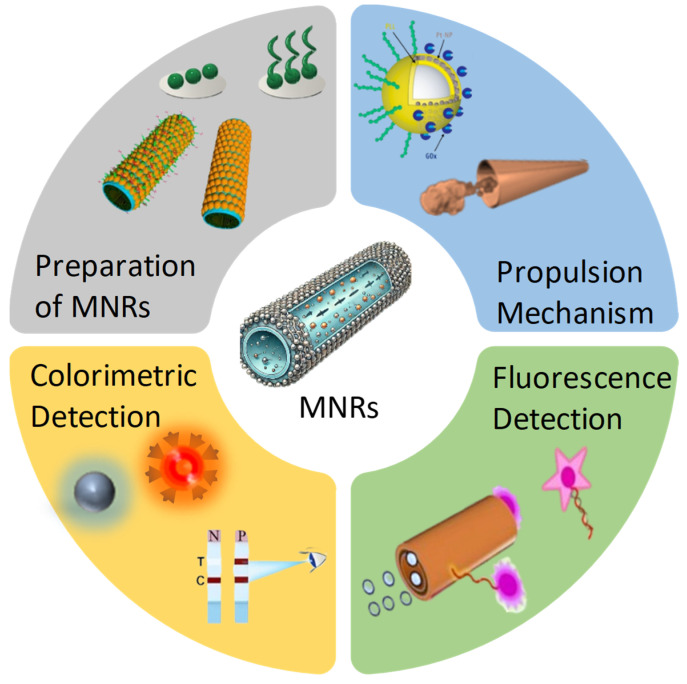
Overview of the preparation, propulsion mechanism, and applications of MNRs.

In recent years, several review articles have highlighted the significant advancements in the sensing and biosensing applications of MNRs [1,2,3,7,8,9,10,11,14,15,16,17,18,19]. Based on their sensing mechanisms, MNR sensors can be broadly classified into three main categories: sensing based on MNR motion; electrochemical sensing with MNRs; and fluorescence, electrochemiluminescence (ECL), and colorimetric sensing using MNRs [1,2,3,7,8,9,10,11,14,15,16]. Sensing mechanisms based on the motion of MNRs are predominantly driven by variations in catalyst activity or fuel availability. Consequently, alterations in the motion of the MNRs can be effectively monitored to detect specific analytes in solution. Similarly, the stirring effect generated by the movement of MNRs can be quantified through electrochemical changes occurring at the electrode surface, thereby enhancing the precision and reliability of the detection outcomes [20,21,22]. In the context of fluorescence, ECL, and colorimetric sensing, the dynamic motion of MNRs facilitates rapid identification of changes in fluorescence, ECL, or colorimetric signals induced by target molecules [2,16,20,21,23,24]. This capability enables highly sensitive and visually interpretable detection, highlighting the efficacy of MNRs as advanced sensing platforms for various analytical applications. Notably, as platforms for “chemistry-on-the-fly”, the movement of MNRs in fluidic environments can significantly accelerate reaction processes, which is particularly advantageous for liquid-based sensing applications [10]. In MNR-based biosensors, the signal conversion system transforms the interactions between the biomolecule recognition units and target substances into electrical or optical signals, often utilizing nanomaterials or micro/nanostructures for enhanced sensitivity and specificity. Despite the considerable exploration of MNRs for various biosensing applications, there is limited literature on fluorescence- and colorimetric-based MNRs [2,16,21]. A unique advantage of these MNRs is their capability for dual-mode detection, which can, in some cases, enable real-time tracking and monitoring of multiple biological events in complex biological environments. Consequently, the present review will begin by focusing on various fabrication methods to create MNRs, the most commonly available propulsion techniques used in MNRs, and the applications of MNRs in biosensing, with a particular focus on motion-driven fluorescence and colorimetric detection methods. Moreover, this review explores the interplay between the synthesis and propulsion mechanisms of MNRs in the development of colorimetric and fluorescence detection platforms, emphasizing their role in facilitating precise and active mixing across diverse environments to enhance analyte interactions with sensing probes. The importance of optimization of synthesis and propulsion mechanisms is emphasized for improving the biocompatibility in biological systems, sensitivity, and specificity of MNRs (Scheme 1).

## 2. Preparation of MNRs

In general, depending on the starting precursor, the manufacturing methods for MNRs are of three types: artificial, biological, and biohybrid. Artificial MNRs dominate the field because they are manufactured using a variety of processing methods and materials, resulting in diverse shapes and functionalities. Biological and biohybrid MNRs are primarily composed of natural biological materials that exhibit a high degree of biocompatibility; however, the challenges associated with their fabrication impose significant limitations on their applications. Artificial MNRs are primarily fabricated using top-down and bottom-up strategies. The top-down method typically involves the fabrication of MNRs using three-dimensional printing techniques, which enables the precise realization of specific shapes and geometries. This approach primarily utilizes micro- and nanolithographic techniques that are characterized by high reproducibility, throughput, and precise geometric control [25]. In contrast, bottom-up methods use physical, chemical, electrochemical, and biological methods, such as atomic layer deposition, polymerization, hydrothermal reactions, template-assisted electrochemical deposition, and bio-conjugation [10,23,24]. In recent years, various techniques have been developed and refined to fabricate MNRs, with significant contributions from advances in micro/nanofabrication technologies. Photolithography, along with various deposition and etching techniques, plays a crucial role in the fabrication of MNRs with high-resolution patterning capabilities [10]. In addition, additive manufacturing techniques have emerged as valuable tools that enable the layer-by-layer construction of MNRs with complex geometries [10]. Recent advancements in preparation methods have enabled the transition of MNRs from the micron scale to the nanoscale. Various MNR designs have been developed, including spirals, tubes, rods, needles, Janus structures, and peanut shapes, employing diverse materials and structures to minimize size [25,26,27,28]. Furthermore, the control methods for MNRs have diversified, allowing for two-to-three-dimensional motion and extending from individual to clustered control. The most widely used techniques for preparing MNRs are discussed in detail in the following sections.

### 2.1. Physical Vapor Deposition

**Figure 1:**
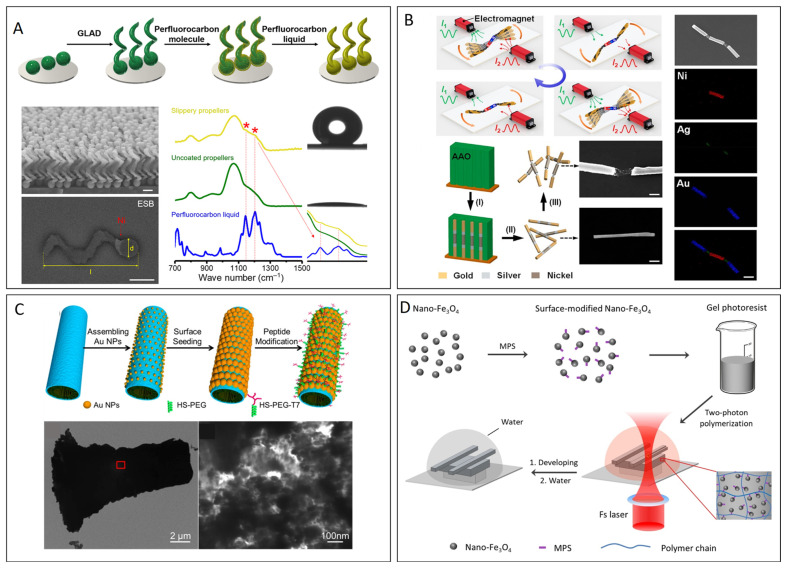
(**A**) Schematic depicting the fabrication process of micropropellers along with the SEM micrographs and FTIR spectral profile of the micropropellers. Reproduced with permission from ref. [32], Copyright 2018, The Authors, American Association for the Advancement of Science. (**B**) Schematic of the synthesis of flexible MNRs and flexible MNRs in an oscillating magnetic field. SEM and EDX images depicting the element distribution of flexible MNRs. Reproduced with permission from ref. [39], Copyright 2021, American Chemical Society. (**C**) Preparation process of PtNP-modified polyelectrolyte multilayer microengines coated with a thin AuNS layer and a tumor-targeted peptide. Reproduced with permission from ref. [44], Copyright 2014, American Chemical Society. (**D**) Synthesis of light-driven hydrogel microactuators. Reproduced with permission from ref. [48], Copyright 2020, Elsevier B.V.

Physical vapor deposition (PVD) is a widely used technique for fabricating MNRs. The versatility of PVD allows precise control over the thickness and composition of the deposited film, making it ideal for producing the functional coatings required for the development of MNRs. This process operates under vacuum and employs physical methods to vaporize the material from a solid or liquid source, converting it into gaseous atoms, molecules, or partially ionized particles. These particles collide, react, and are ultimately deposited on the substrate, creating an interconnected film with specific functions. Ma et al. utilized electron beam evaporation to deposit thin layers of Pt (10–15 nm) and SiO_2_ onto a monolayer of polystyrene particles. This method was used to fabricate the reversed Janus MNRs using an internal chemical engine [29]. In a similar study, Baraban et al. applied sputter coating to deposit multilayered [Co/Pt(Pd)]N metal films on silica surfaces, thereby producing cathodic spherical microrobots. These microrobots demonstrate the ability to move and release drugs in response to an external magnetic field. Huang et al. combined conventional PVD with wet chemical etching to create nanoshell micromotors of varying diameters (2–30 μm). This process involved the sequential deposition of three layers of Pt, Ag, and Au onto silica beads, followed by etching with hydrofluoric acid to remove the silica core and form hollow micromotors [30,31]. A recent advancement in PVD is glancing angle deposition (GLAD), in which the substrate is dynamically manipulated during the deposition process, unlike the stationary approach used in conventional PVD (Figure 1A) [32]. The GLAD process begins with the deposition of the material onto the substrate at a glancing angle. In this approach, vaporized atoms arrive at the substrate surface at an acute angle, resulting in the formation of core nuclei that are initially deposited in a slanted manner [32,33,34]. As the core nuclei grow, they begin to obstruct the path of the incoming vapor, leading to the formation of shadows on the substrate. With continued deposition, the initially slanted nuclei evolved into elongated columns. These columns increase in height and inhibit further deposition in the shadowed regions, leading to a columnar structure. In the final stage, the columns continue to grow at tilted angles. This stage is marked by ongoing competition between direct deposition on the exposed substrate areas and the shadowing effect created by the growing columns. The resulting highly porous and anisotropic structure is characterized by well-defined features that can be tuned for specific applications. By rotating the substrate at a constant rate after depositing the seed layer, the vertical structures can be shaped into helical forms. This method is particularly effective for fabricating helical magnetic MNRs and for providing precise structural control, which is essential for various applications in robotics. Rod-shaped MNRs were fabricated using the GLAD–dynamic shadowing growth technique, where a 2 μm TiO_2_ arm was deposited on 1 μm silica (SiO_2_) spheres. Under low-intensity UV light in an aqueous peroxide medium, these MNRs exhibit self-propulsion, with swimming behavior influenced by medium pH [35]. Srikanta et al. developed TiO_2_/Cu_2_O–silica Janus MNRs capable of self-propulsion under low-intensity light in aqueous peroxide. These micromotors were functionalized with fluorescent dyes, including Alq_3_, Alizarin, and zinc phthalocyanine, using the GLAD technique. The capping of dye molecules over the surface of MNRs makes them ideal candidates for colorimetric or fluorescence detection applications [36]. However, despite its utility in these applications, PVD has certain limitations. One major drawback is the necessity for an ultraclean vacuum environment during the process, which increases the operational complexity and costs [31].

### 2.2. Template-Assisted Electrodeposition Technology

Electrodeposition has become a popular method for fabricating MNRs because of its low cost, high efficiency, and precise control [10,31]. In particular, template-guided electrodeposition is widely used in the preparation of MNRs, in which an electric current is applied to reduce metal cations or conductive polymers on the surface of an electrode covered by a porous filter membrane [10]. This membrane restricted the growth of the deposited material within its pores. By controlling the pore size of the membrane, it is possible to design the size of the resulting MNRs. Once the deposition process is complete, the membrane is dissolved, releasing the fabricated robots into a solution [37]. Paxton et al. were the first to successfully fabricate nanomotors by electrodeposition. They prepared Pt/Au nanorods with diameters of approximately 370 nm consisting of alternating platinum and gold segments [38]. The developed nanomotors demonstrated directional movement when placed in aqueous H_2_O_2_ solutions, exhibiting non-Brownian motion. To enhance the speed and efficiency of MNRs, researchers have replaced the gold segments with Ag/Au alloys. This modification resulted in an improved propulsion performance. Moreover, the addition of carbon nanotubes (CNTs) to the Pt segment enhances the propulsion capabilities of the nanomotors. Ji et al. recently developed flexible MNRs capable of exhibiting a swinging motion. These nanomotors were fabricated using a sequential, part-by-part electrodeposition process involving the deposition of Au, Ag, and Ni layers on an aluminum oxide membrane. This approach allows the creation of flexible nanomotors with unique motion characteristics (Figure 1B) [39]. It has been well documented that tubular MNRs achieve propulsion speeds of hundreds of μm s^−1^ through H_2_O_2_ under a suitable metal catalyst (Pt). In general, tubular MNRs are fabricated via sequential electrodeposition of sensing substrates such as reduced graphene oxide (rGO) or graphene quantum dots (GQDs) and Pt using a nanoporous membrane template [40]. For example, MNRs functionalized with fluorescein amidine (FAM)-tagged DNA have been used for sensing applications [41].

### 2.3. Self-Assembly

Self-assembly is a fundamental phenomenon in which basic structural units spontaneously organize or aggregate into stable structures characterized by regular geometrical features [42]. This process is primarily driven by noncovalent bonding interactions such as van der Waals forces, hydrogen bonding, and electrostatic interactions [43]. For example, layer-by-layer self-assembly involves the sequential deposition of charged materials to form multilayer membranes. During this process, oppositely charged polymer anions and cations are alternately deposited, leading to the formation of a stable supramolecular structure [43]. He et al. employed a layer-by-layer self-assembly technique to prepare multilayer hollow asymmetric MNRs using Pt nanoparticles. The resultant unique multilayer structure, along with the excellent loading capacity of these MNRs, renders them suitable for various biomedical applications such as targeted drug delivery and imaging. Magnetic-field-induced self-assembly utilizes the versatility of magnetic forces to structure materials. This technique is particularly advantageous for fabricating swarm robots, owing to its ability to facilitate controllable structural reconfiguration (Figure 1C) [44]. For example, Tasci et al. utilized a two-dimensional rotating magnetic field to develop superparamagnetic beads capable of self-assembling into size-controlled microwheels [45]. The application of the rotating magnetic field allows the manipulation of these beads, resulting in the formation of well-defined structures that can be engineered for specific functionalities. Wang et al. developed tubular fluorescent MNRs using PEDOT/PSS/Pt bilayers by exploring the electrostatic self-assembly of CdTe QDs. The layer-by-layer modification yielded a highly fluorescent surface. The MNRs powered by 5 wt% H_2_O achieved speeds of 201 ± 52 μm/s through bubble propulsion. The integration of CdTe QDs facilitated real-time optical detection via fluorescence quenching upon analyte binding [46]. In a recent work, Sánchez et al. used a self-assembly method for scalable production of platinum and iron oxide nanoparticle-based MNRs, functionalized with phenylboronic acid-modified GQDs, emitting blue fluorescence. The prepared MNRs were successfully employed for the sensitive fluorometric detection of endotoxins [47]. Although self-assembly techniques are valuable because of their ability to create complex structures through bottom-up approaches, they involve time-consuming processes and can produce a restricted range of shapes, which are significant challenges that must be addressed for the broader adoption of these techniques in the field of MNRs.

### 2.4. Direct Laser Writing

Direct laser writing (DLW), which uses two-photon polymerization (TPP) nanolithography, has been widely applied to the fabrication of three-dimensional (3D) microstructures with high spatial resolution [33,48]. DLW via TPP employs a high-intensity femtosecond laser that emits extremely short pulses of light. This laser selectively polymerizes photosensitive materials along a preprogrammed 3D path through a nonlinear absorption mechanism. Laser light causes chemical reactions in a material at specific locations, enabling the creation of complex structures with high precision. After the laser writes the desired pattern, the next step involves developing a material similar to that used in traditional photolithography. Depending on the type of photosensitive resin used, areas that were either exposed or unexposed to the laser can be selectively removed using a developer solution. Although DLW is generally slower in terms of fabrication speed compared to other 3D printing methods, its primary advantage lies in its superior resolution, which can achieve feature sizes as small as 100 nm (Figure 1D) [33,48]. Dong et al. employed TPP-based 3D laser lithography in conjunction with water dispersion to successfully fabricate helical microstructures composed of biodegradable soft hydrogels [49]. Following the fabrication of helical microstructures, the researchers coated these structures with magnetoelectric nanoparticles, which enabled the helical microstructures to be activated by an external rotating magnetic field, thus facilitating controlled movement. Suter et al. proposed the development of a spiral microrobot using 3D printing technology made from a magnetic polymer composite (MPC), which consists of magnetite (Fe_3_O_4_) nanoparticles embedded in SU-8 resin [50]. The embedded magnetic nanoparticles allowed the robot to execute corkscrew motion when subjected to a rotating magnetic field, enabling it to swim with precise control. Li et al. used 3D laser lithography to create a microrobot with a burr-like porous spherical structure [51]. The functionality of the microrobot was further enhanced by coating its surface with Ni and Ti. This coating enabled the microrobot to effectively carry and deliver drugs to target cells in vivo (within a living organism), driven by an external magnetic field. Nelson et al. developed artificial bacterial flagella (ABFs) functionalized with near-infrared fluorophore NIR-797 using 3D-DLW and TPP [52]. The helical structures of ABFs were coated with 50 nm of Ni for magnetic control, while the NIR-797 fluorophores were attached via an aminosilane layer and 5 nm of Ti for biocompatibility. Under a controlled magnetic field, ABFs achieved stable translational motion at an average speed of 70.4 μm/s. Their long-wavelength emission was explored for fluorescence imaging applications. In a recent study, Ren et al. developed a TPP strategy to fabricate ant microbots composed of magnetic photoresist, hydrogel, and metal nanoparticles [53]. These microbots were shown to selectively and reversibly assemble into various morphologies under combined magnetic and light fields, suggesting their potential in drug delivery and sensing applications.

### 2.5. Microfluidics

**Figure 2:**
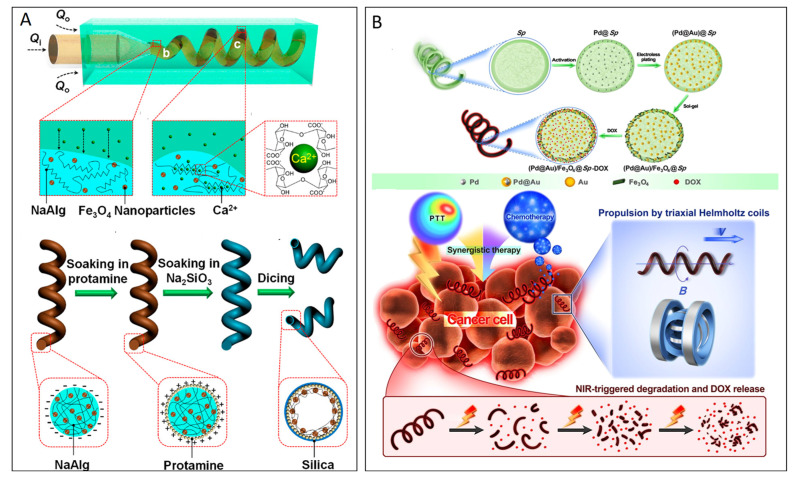
(**A**) Scheme depicting the microfluidics-assisted fabrication of magnetic hybrid microswimmers with hollow helical structures. Reproduced with permission from [60], Copyright 2018, American Chemical Society. (**B**) Fabrication of (Pd@Au)/Fe_3_O_4_@Sp.-DOX MNRs and schematic of propulsion, NIR-triggered degradation, and DOX release for chemo-photothermal cancer therapy. Reproduced with permission from [62], Copyright 2019, American Chemical Society.

Microfluidics has been proven to be an ideal technique for the controlled synthesis of nanoparticles [54,55]. As compared to conventional preparation methods, the use of microfluidics offers several advantages for preparing MNRs with adjustable sizes and compositions and high uniformity. The microfluidic technique typically makes use of the viscosity difference between the inner and outer fluids. Beyond a specific threshold where the viscosity of the inner fluid exceeds that of the outer fluid, the “liquid rope-coil effect” is triggered, leading to the formation of helical MNRs [56,57]. For instance, Yu et al. demonstrated the preparation of helical MNRs by accurate control of fluid dynamics within microfluidic channels, coupled with a dicing process. They showed that by modulating flow rates and cutting distances, the morphology of magnetically responsive helical MNRs can be fine-tuned [58]. In a recent work, Dong et al. utilized a “microscale liquid rope-coil effect” strategy to fabricate graphene oxide-based helical magnetic MNRs in glass capillary microfluidics. The prepared MNRs, along with Ag-modified MNRs, have been effectively utilized for water remediation, demonstrating exceptional efficiency in removing both chemical and biological contaminants. Additionally, the functionalization of GFHMs with doxorubicin has been successfully explored for drug delivery applications [59]. Tang et al. fabricated magnetic hybrid microswimmers from microfluidic coiled flows by preparing Fe_3_O_4_ nanoparticles comprising helical Ca-alginate microfibers. Finally, the hollow helical structures were achieved via a controllable dicing and biosilication process (Figure 2A) [60].

### 2.6. Biohybrid MNRs

The fabrication of MNRs with an ideal morphology and biocompatibility for use in living organisms remains challenging. Recently, biohybrid MNRs have successfully merged living microorganisms with synthetic micro/nanomaterials to produce highly functional systems [33,61]. These inherent biological properties enable robots to precisely navigate complex and dynamic environments in response to chemical and physical stimuli. When biomolecules are integrated with magnetic particles, polymers, or nanowires, the resulting system can be externally controlled through stimuli, such as magnetic fields or light, enhancing their functionality and directionality [33,61]. For example, Wang et al. used the microalga Spirulina (Sp.), which has a distinctive 3D helical structure, as a biotemplate to create biohybrid helical MNRs for cargo delivery and chemo-photothermal therapy (Figure 2B) [62]. They showed that microalgae-based magnetic microrobots achieved impressive swimming speeds of 526.2 μm/s when subjected to a rotating magnetic field. Veronka et al. demonstrated a single-step electrostatic self-assembly technique to fabricate IRONSperms, which are soft magnetic microswimmers that mimic sperm cells [63]. Their study revealed that these microswimmers exhibit a swimming speed greater than 0.2 body lengths per second, with an average velocity of 6.8 ± 4.1 µm/s. IRONSperms developed under magnetic actuation were successfully applied for in vivo targeted therapy. Dong et al. developed fluorescent micromotors by incorporating catalase (CAT) into polycaprolactone (PCL) to form PCL-CAT micromotors, followed by immobilization of fluorescein isothiocyanate (FITC) via isothiocyanate–thiol reactions [64]. The catalase, containing porphyrin heme groups, enables self-propulsion by decomposing H_2_O_2_ into water and oxygen. Under 488 nm excitation, the micromotors emit green fluorescence, with intensity modulated by exposure to hydrogen chloride or ammonia gases. In another work, NIR-propelled MNRs for enzyme-based colorimetric detection of cholesterol were developed by functionalizing cholesterol oxidase (ChOx) Fe_3_O_4_ nanoparticles (~2 nm). The ChOx enzyme oxidized cholesterol to produce H_2_O_2_, while NIR exposure created a photothermal gradient in Fe_3_O_4_ nanoparticles [65]. The enzymatic and magnetic–photothermal processes enabled dual propulsion of the MNRs along with the colorimetric readout for cholesterol detection.

## 3. Propulsion Mechanisms

MNRs can be engineered using synthetic components or combinations of synthetic and biological elements. Their propulsion is typically driven by chemical fuels, external fields (e.g., magnetic, electric, or light), or biological forces, enabling efficient movement in various fluidic environments. In the subsequent sections, a detailed discussion is provided on MNRs, focusing on their energy sources and propulsion mechanisms and highlighting the distinct advantages and challenges associated with each approach.

### 3.1. Chemical Propulsion

Chemically powered MNRs are a class of autonomous machines capable of harvesting energy directly from chemical fuels present in their environment, such as H_2_O_2_, N_2_H_4_, urea, or glucose. These MNRs utilize chemical reactions to achieve propulsion, often through the asymmetric distribution of reaction products across their surfaces. The self-propulsion of these MNRs is primarily based on four key mechanisms: self-electrophoresis, self-diffusiophoresis, and bubble propulsion [2,22]. Self-electrophoretic propulsion is a mechanism in which propulsion is generated by the interaction between the charged surface of an MNR and the electric field produced by the MNR via asymmetric chemical reactions. Unlike traditional electrophoresis, which relies on an external electric field to induce motion, self-electrophoresis involves a self-sustained process in which oxidation and reduction reactions occur in different regions of the MNR, typically on Janus particles. These reactions lead to the uneven generation and consumption of charged species, creating a local electric field around the particle. The interaction between this self-generated electric field and the charged surface of the MNR induces electrokinetic flow in the surrounding fluid, driving autonomous motion in the particle [66]. The catalytic decomposition of H_2_O_2_, along with various other redox reactions involving chemicals such as N_2_H_4_ and Br_2_/I_2_, serves as a prominent example of self-electrophoresis-based propulsion in MNRs, particularly Janus particles [67].

**Figure 3:**
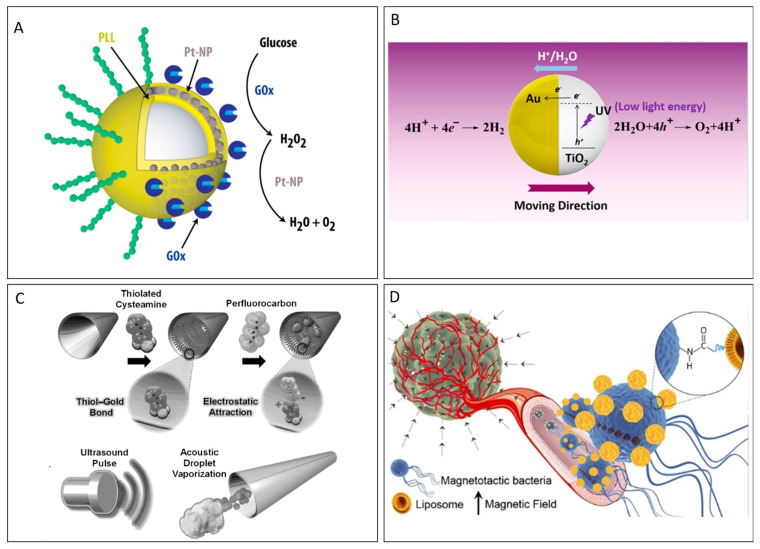
(**A**) GOx/Pt-NP swimmer. Reproduced with permission from [70], Copyright 2017, American Chemical Society. (**B**) Schematic of catalytic TiO_2_–Au Janus micromotors powered by UV light in water. Reproduced with permission from [73], Copyright 2015, American Chemical Society. (**C**) Preparation of PFC-loaded MBs. Reproduced with permission from [78], Copyright 2012, The Authors, Published by WILEY-VCH Verlag GmbH & Co. (**D**) Magnetotactic bacteria. Reproduced with permission from [83], Copyright 2014, American Chemical Society.

In self-diffusiophoresis, propulsion relies on asymmetric chemical reactions that typically occur on one side of Janus particles [2,68]. The propulsion mechanisms in self-diffusiophoresis can be further categorized into two types based on the nature of the reaction products: nonelectrolyte and electrolyte diffusiophoresis [69]. In the case of nonelectrolyte diffusiophoresis, the reaction products are neutral, and no electric field is generated. Instead, propulsion arises because of the interaction between the product molecules and the particle via nonelectrostatic forces such as van der Waals forces or excluded volume effects. The strength and direction of the propulsion are determined by the differences in how the product molecules interact with different regions of the particle surface. These nonelectrostatic forces influence the local concentration gradients around the particle and drive its motion. In contrast, electrolyte diffusiophoretic propulsion occurs when chemical reactions produce ionic species. These ions diffuse at different rates around the particles, creating a local electric field. This electric field couples with the surface charges of the particles, inducing an electrokinetic flow in the surrounding fluid, which propels the MNR. For instance, the ability to dynamically control the swarm behavior of Au microparticles through chemical gradients suggests the potential of self-diffusiophoretic systems for coordinated collective motion in MNR applications. Recently, remotely controlled Janus particles using two biological fuels, glucose oxidase and catalase, have been reported. Enzymatic reactions generate concentration gradients of the reaction products around the particles, resulting in self-diffusiophoresis, which facilitates the controlled motion of Janus particles (Figure 3A) [70].

In bubble-propelled MNRs, gas bubbles are generated when the volume of oxygen produced from the catalytic decomposition of H_2_O_2_ exceeds the local solubility limit. Upon detachment, these bubbles transfer momentum to the MNR and propel it in the opposite direction. Notably, the earliest instance of bubble propulsion involved the catalytic decomposition of H_2_O_2_ using Pt, which produced oxygen to propel a robot [71]. Recently, magnesium has gained attention as a promising material for constructing bubble-propelled micromotors owing to its moderate reaction rate with water. For example, in the presence of a NaHCO_3_ aqueous solution, the surface passivation layer of Mg(OH)_2_ dissolves quickly, forming soluble MgCO_3_ and continuously exposing the active Mg surface. This results in the generation of hydrogen bubbles from the reaction between Mg and water, which propel the micromotors [72].

### 3.2. Physical- or External-Field-Controlled MNRs

Physical- or external-field-controlled MNRs involve controlled locomotion through the application of external physical fields, including light, ultrasound, magnetic, and electric. Light-propelled MNRs are engineered using photoresponsive materials, including photochemical, photothermal, and photoisomerized substances [20,22]. For example, Dong et al. demonstrated the photocatalytic propulsion of Au-TiO_2_ Janus micromotors driven by UV light (Figure 3B) [73]. Researchers have explored the use of visible light in combination with photocatalytic metal-oxide-incorporated Janus CuO-Au microparticles to generate motion in H_2_O_2_ solutions [74]. This approach utilizes the photocatalytic properties of metal oxides within the Janus structure, enabling efficient propulsion mechanisms under visible light illumination. Recently, photochemical and photothermal MNRs have garnered significant attention owing to their capacity to generate stronger propulsion than other light-driven systems. For instance, propulsion in photothermal MNRs occurs via self-thermophoresis. This process occurs when near-infrared (NIR) light illuminates the surfaces of Janus particles with a high extinction coefficient [75]. Consequently, this illumination creates an asymmetric temperature distribution in the surrounding fluid, leading to autonomous motion of the particles. Common examples include dielectric microspheres comprising a thin layer of gold or carbon. The temperature difference generated between the two layers under NIR light acts as a driving force for the particles [76].

Ultrasound is a noninvasive method for the on-demand motion control of MNRs, enabling sustained operation while ensuring favorable biocompatibility. The use of ultrasound for propulsion allows MNRs to navigate complex biological environments effectively, making them particularly suitable for biomedical applications. By harnessing ultrasonic waves, these MNRs can achieve precise movement and manipulation within various biological media, which is essential for tasks such as targeted drug delivery, minimally invasive surgery, and diagnostic procedures [2,77]. Wang et al. demonstrated that ultrasound can vaporize fuel within micromachines, resulting in significant momentum generation. This enhanced propulsion enables the micromachines to penetrate biological tissues at high velocities, demonstrating their potential for effective navigation in biomedical applications (Figure 3C) [22,78].

Magnetic actuation is a widely used technique for controlling the movement of MNRs owing to its noninvasive nature and compatibility with biological systems [2,3,31]. This method utilizes magnetic fields to exert forces on the magnetic particles embedded within robots, enabling propulsion. Typically, magnetic MNRs operate effectively in low-Reynolds-number fluids, where viscous forces dominate inertial forces [2,3,31]. One of the primary challenges associated with magnetic-field-induced propulsion is the design of both magnetic fields and magnetic particles. Achieving precise control over the movement of the MNRs requires careful consideration of these parameters to ensure accurate navigation. For example, by employing rotating magnetic fields, researchers have successfully developed helical robots capable of propulsion through various induced motions, including rolling, corkscrew, and spin-top movements [79]. In addition to magnetic-field-induced propulsion, MNRs can be effectively controlled using external electric fields, which enable the precise manipulation of their movement through various electrokinetic phenomena, such as electro-osmosis and electrophoresis [2,3,22]. Electro-osmosis refers to the flow of liquid through a porous surface induced by the application of an electric potential. This technique allows for the controlled movement of fluids in micro/nanoscale environments, facilitating tasks such as transport and mixing within confined spaces. Electrophoresis involves the migration of charged particles through a liquid or gel when subjected to an electric field. Furthermore, direct current fields have been effectively utilized to drive charged particles or molecules through electrophoresis, while dielectrophoretic forces generated by nonuniform electric fields can also propel MNRs. Additionally, asymmetric particles subjected to alternating current fields can move through mechanisms such as induced-charge electrophoresis and electro-osmotic propulsion [80,81].

### 3.3. Biologically Propelled MNRs

To address the biocompatibility challenges associated with synthetic MNRs, recent research has focused on the development of biohybrid MNRs that combine artificial and biological components to mimic living organisms. The biological components of biohybrid robots, such as bacteria, sperm cells, algae, neutrophils, and macrophages, provide natural propulsion mechanisms and biological functions such as chemotaxis, phototaxis, and magnetotaxis [2,22,33]. For example, sperm cells, known for their role in reproduction, exhibit strong chemotactic behavior as they swim toward egg cells, making them highly effective in directed motion [2,22]. Schmidt et al. demonstrated the development of a hybrid sperm MNR capable of active locomotion against blood flow, facilitating the targeted delivery of heparin cargo [82]. In contrast, bacteria possess natural environmental sensing abilities that allow them to respond to various stimuli such as chemoattractants, pH, oxygen levels, temperature, and magnetic fields (Figure 3D) [2,83]. Neutrophils, a type of white blood cells, respond rapidly to inflammation by migrating through endothelial tissues to sites of inflammation. Zhang et al. developed “neutrobots” by employing natural neutrophils to phagocytose drug-loaded magnetic nanogels enveloped in *Escherichia coli* membranes [84]. They showed that upon exposure to a rotating magnetic field, the neutrobots exhibited controllable intravascular movement, enabling them to autonomously aggregate in the brain and cross the blood–brain barrier via positive chemotaxis along inflammatory gradients. As a result, neutrophil-based MNRs have been employed to deliver therapeutic nanoparticles across endothelial barriers, specifically targeting inflamed or diseased tissues [84].

### 3.4. Importance of Propulsion Mechanisms in MNRs for Colorimetric and Fluorescence Sensing Applications

The use of an appropriate propulsion mechanism in MNRs is crucial for enhancing the efficiency and precision of colorimetric and fluorescence sensing applications. A propulsion mechanism enables MNRs, unlike conventional sensing probes, to actively mix within diverse environments including heterogeneous media, biological fluids, and microchannels. For instance, the high mobility offered by chemical propulsion enables MNRs to actively mix with the surrounding medium, promoting efficient interactions between target analytes and sensing components such as dyes, enzymes, or nanoparticles, thereby enhancing detection sensitivity [2]. In chemical propulsion, sustained motion is achievable as long as the fuel supply is maintained, mitigating concerns associated with external field safety. However, the potential toxicity of the commonly used fuels (e.g., H_2_O_2_) and their byproducts (e.g., ROS and metal ions) must be carefully evaluated, particularly for long-duration sensing applications [16]. For instance, it is crucial to ensure the biodegradation of MNRs without the accumulation of harmful residues and to mitigate the risk of fuel depletion, which could interrupt propulsion. Physical fields, such as magnetic and ultrasound-driven propulsion mechanisms, are highly effective in fluidic environments and exhibit excellent compatibility with biological systems. These approaches are noninvasive, controllable, and well suited for sensitive biological applications, offering precision and safety in diverse operational contexts. Light-driven MNRs offer advantages such as compatibility with a wide range of light-responsive, biocompatible, and biodegradable materials, along with higher energy efficiency due to tunable optical absorption. These features make them promising for sensing applications [2]. However, light-driven propulsion mechanisms have limitations which include restricted penetration depth as well as constraints on speed and output force. To overcome penetration challenges, optical-fiber-based systems could enhance MNRs’ applicability for deeper tissue sensing. In contrast to other propulsion mechanisms, biologically propelled MNRs effectively navigate complex environments, enabling targeted mobility crucial for sensing applications with low or unevenly distributed analytes [16]. In colorimetric sensing, this allows localization in chemical gradient regions, enhancing analyte accumulation and detection. Thus, the nature of the propulsion mechanism in MNRs plays a vital role in optical detection applications.

## 4. Applications of MNRs in Colorimetric and Fluorescence Sensing

It is well known that surface-based sensing relies on the stationary capture of target analytes in liquid or gas phases by surface-functionalized probes and is influenced by two primary factors: the intrinsic chemical reaction kinetics of the probe–target interaction and the physical transport efficiency of analytes to the reactive surface [10,85]. The former encompasses various recognition mechanisms such as electrochemical transduction and affinity-based reactions, whereas the latter involves diffusion and convection as the key drivers of analyte transport. However, at ultralow concentrations, mass-transport limitations in steady-state systems or microfluidic environments pose significant challenges, leading to reduced sensitivity and increased response times. In contrast, MNRs, which serve as mobile platforms for “chemistry-on-the-fly”, offer innovative solutions by actively enhancing analyte recognition and mass transport [11]. Their motion not only responds to environmental changes, offering real-time detection capabilities, but also induces fluid convection and mixing, significantly improving the transport efficiency [11,85]. This dynamic interaction between the motion and mass transport in MNRs helps overcome the limitations of traditional surface-based sensing, resulting in faster response times and improved detection sensitivity. Consequently, MNRs have been used in several sensing applications, including electrochemical, colorimetric, fluorescence, and motion-based mechanisms. Recently, MNRs have emerged as powerful tools for sensing applications, particularly because of their unique motion-driven signal transduction mechanisms. Among the most notable advancements is the use of MNRs in colorimetric and fluorescence-based detection systems [10,16,21]. Using the controlled motion of MNRs, it is possible to induce dynamic changes in fluorescent signals or colorimetric responses, leading to enhanced sensitivity and selectivity in the detection of biomolecular interactions.

### 4.1. Detection Principles in MNRs

The most commonly used colorimetric/fluorescence detection principles in MNRs are classified into two types, namely, homogeneous and heterogeneous assays. Homogeneous assays involve simple mixing of probes and analytes, where target detection is based on signal changes [86]. On the contrary, heterogeneous assays utilize immobilized components on solid supports, allowing for enhanced separation of bound and unbound reagents and reducing background interference, thereby improving detection specificity and accuracy. In general, colorimetric sensing relies on visual changes, often in color, to detect target analytes. The presence of the target molecule is ascertained by measuring the changes in the signal of the probe [86]. The colorimetric detection principles in homogeneous assays mostly involve the use of enzymes or enzyme mimics for the colorimetric changes of a substrate in the presence of MNRs. For instance, enzyme-propelled magnetic MNRs have been employed for colorimetric sensing by making use of enzymatic reactions to drive their propulsion and facilitate the detection of target analytes through observable color changes. MNRs functionalized with antibodies have been examined in both static and dynamic fluidic environments, demonstrating potential for practical application in heterogeneous assays [16]. Commonly used fluorescence detection mechanisms in MNRs include Förster resonance energy transfer (FRET), immunoassays/aptassay, DNA hybridization, and chelation-induced fluorescence changes. Based on the recognition elements used such as dye-tagged DNA or dye-labeled MNRs, these assays are classified as homogeneous or heterogeneous. The nanoprobes that utilize FRET and interactions driven by fluorescence turn-on or turn-off mechanisms fall under the classification of homogeneous assays. MNRs functionalized with dye-labeled probes have been applied in FRET-based fluorescence assays. In this, the MNRs act as a fluorescence-quenching platform through π–π stacking interactions with the dye fluorophores. Upon the introduction of target analytes, such as complementary DNA sequences, these interactions are disrupted, leading to a fluorescence turn-on signal [16].

### 4.2. Colorimetric Sensing Using MNRs

The development of MNR-based biosensors that detect analytes based on changes in movement and electrical signals has resulted in significant advancements. However, Alberto et al. demonstrated the first practical application of these systems for biosensing purposes using millimeter-sized tubular mobile motors [87]. These motors were self-propelled by the surface-induced Marangoni effect facilitated by the simultaneous release of sodium dodecyl sulfate and horseradish peroxidase (HRP). The catalytic activity of HRP enabled the detection of H_2_O_2_ via electrochemical, optical, and even visual methods, underscoring its versatility. In contrast to earlier micromotor-based immunosensing strategies, which typically relied on direct or sandwich immunoassays with microsphere tracers for visualizing antibody–antigen interactions, Berta et al. developed a competitive immunoassay for the naked-eye detection of cortisol [88]. The integration of specific antibodies with the efficient movement of H_2_O_2_-propelled tubular micromotors enabled the rapid detection of dilute cortisol–HRP levels using minimal microliter-scale sample volumes. They showed that the micromotor-based sensing technique allows specific detection of cortisol down to 0.1 µg mL^−1^, offering significant potential for rapid, point-of-care diagnostics [88]. Roberto et al. reported the synthesis of single-wall carbon nanotube–Fe_2_O_3_/MnO_2_ micromotors for the detection and discrimination of phenylenediamine isomers in water samples [89]. These tubular micromotors were constructed with a hybrid outer layer comprising single-wall carbon nanotubes and Fe_2_O_3_ nanoparticles, powered by a MnO_2_ catalyst. The catalytic decomposition of H_2_O_2_ served as fuel, generating oxygen bubbles and hydroxyl radicals that facilitated the dimerization of phenylenediamine isomers, resulting in distinct color changes in colorimetric assays.

**Figure 4:**
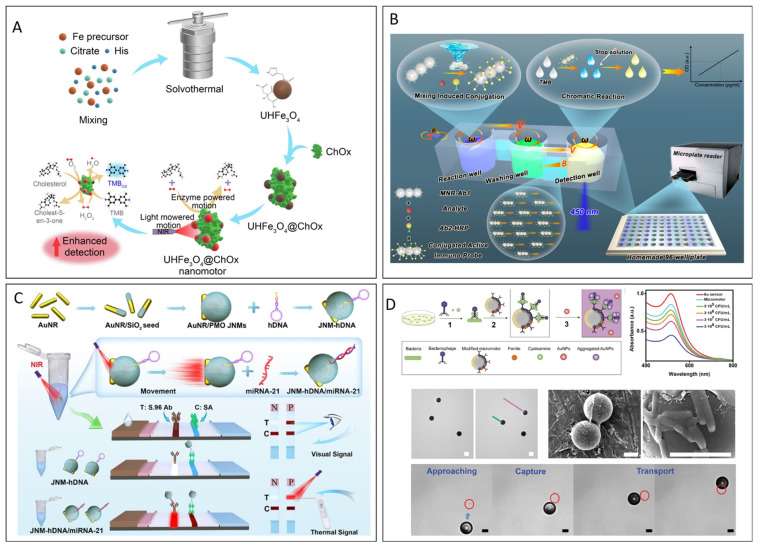
(**A**) Schematic illustrating the fabrication of a double-engine micromotor and its application for colorimetric cholesterol detection. Reproduced with permission from [65], Copyright 2022, Elsevier B.V. (**B**) Magnetic nanorobot-enabled automated and efficient ELISA (nR-ELISA) analysis. Reproduced with permission from [90], Copyright 2022, American Chemical Society. (**C**) Schematic of the synthesis process of AuNR/PMO JNMs and principle of the AuNR/PMO JNM-assisted LFTS for the rapid dual-modal and amplification-free detection of microRNA. Reproduced with permission from [93], Copyright 2023, American Chemical Society. (**D**) Schematic of bacteriophage-functionalized magnetic Janus micromotors for E. coli bacteria biosensing. Reproduced with permission from [95], Copyright 2024, WILEY-VCH Verlag GmbH & Co.

Recently, an enzyme/light-powered nanomotor was developed for the rapid and sensitive detection of cholesterol utilizing cholesterol oxidase (ChOx) as the model enzyme (Figure 4A) [65]. This system involved the co-assembly of ChOx with ultra-small histidine-modified Fe_3_O_4_ nanoparticles (UHFe_3_O_4_). The UHFe_3_O_4_@ChOx nanomotor operates as a mobile biosensor, exhibiting concentration-dependent self-propulsion using cholesterol as fuel and NIR-powered directional movement, facilitated by the photothermal properties of UHFe_3_O_4_ nanoparticles. The system makes use of the chromogenic reaction between ChOx and UHFe_3_O_4_, enabling real-time colorimetric analysis of cholesterol. To address the limitations of conventional methods, MNRs have been developed as controllable probes for enzyme-linked immunosorbent assays (ELISAs). For instance, rod-like MNRs, constructed from Fe_3_O_4_@SiO_2_ core–shell nanorods with a one-dimensional (1-D) morphology, have been modified with a capture antibody (Ab1) to create movable immunoassay probes (MNR-Ab1s) [90]. In the presence of a gradient and rotating magnetic field, MNR-Ab1s can be precisely directed to the target sites and effectively mixed, facilitating homogeneous reactions. The unique 1-D structure of the rotating MNR-Ab1s enhances fluid stirring, significantly reducing the incubation time and improving the detection efficiency in immunoassays (Figure 4B) [90]. Guo et al. demonstrated the potential of nanomotors as active probes for lateral flow immunoassays (LFA) by synthesizing Au@mSiO_2_@Pt Janus nanomotors [91]. In this system, a Pt nanolayer was deposited on one-half of the Au@mSiO_2_ nanoparticles, enabling the catalytic decomposition of H_2_O_2_ to generate propulsion. The motion characteristics of the Au@mSiO_2_@Pt nanomotor in static fluidic environments and dynamic flow fields were optimized to facilitate its application in LFIA. The nanomotor was subsequently functionalized with antibodies and used as an active immunoassay probe to target pepsinogen I (PG I) and pepsinogen II (PG II). The results indicated that, compared to traditional Au nanoprobes, the nanomotor-based probes significantly enhanced sensitivity by improving the binding efficiency between antigens and antibodies, achieving limits of detection (LODs) of 2.2 ng/mL for PG I and 2.1 ng/mL for PG II. A metal–organic-framework-based MIL-88B@Fe_3_O_4_ micromotor has been developed for the colorimetric detection of phenolic compounds in microdroplets. The developed system utilizes spindle-like MIL-88B integrated with doped Fe_3_O_4_ in conjunction with phenolic compounds, 4-aminoantipyrine (4-AAP), and H_2_O_2_ to facilitate colorimetric detection [92]. Chen et al. reported the development of NIR-powered Janus nanomotors composed of Au nanorods and mesoporous organo-silica microspheres (AuNR/PMO JNMs) as “swimming probes” to enhance lateral flow test strips (LFTSs) for the quantitative detection of miRNA-21 in serum and cell culture media (Figure 4C) [93]. Detection was achieved by conjugating AuNR/PMO JNMs with specifically designed hybrid DNA as recognition probes for miRNA-21. Upon NIR irradiation, the AuNRs generated asymmetric thermal gradients around the JNMs, resulting in vigorous self-propelled thermophoretic motion that significantly accelerated the recognition of miRNA-21 targets, allowing for the direct quantitative detection of miRNA-21 on the LFTS, producing both visual and thermal signals. The AuNR/PMO JNMs showed a limit of detection of 18 fmol/L for miRNA-21 and were successfully used to detect miRNA-21 in spiked serum samples and MDA-MB-231 cell culture media. Yang et al. developed a bioinspired hierarchical ZrO_2_/MnFe_2_O_4_/FeZr-MOF tubular magnetomotor with intrinsic peroxidase-like activity, serving as an advanced platform for simultaneous colorimetric detection and specific purification of trace levels of glyphosate in complex environmental matrices under neutral conditions [94]. The engineered Zr-based micromotors consisted of ZrO_2_ microtubes for structural support, MnFe_2_O_4_ nanosheets as catalysts and magnetic guides, and FeZr-MOF octahedrons exhibiting peroxidase-like activity, resulting in a unique hierarchical architecture with abundant reactive sites. These chemically powered microrobots demonstrated autonomous circular motion, propelled by O_2_ bubbles generated from the MnFe_2_O_4_-catalyzed decomposition of H_2_O_2_ at a velocity of 101.6 ± 10.2 μm s^−1^ in 5% H_2_O_2_. The combination of autonomous movement, strong affinity between Zr–O clusters and phosphate groups, and robust peroxidase-like activity enabled the Zr-based micromotors to effectively capture and adsorb glyphosate, achieving a limit of detection (LOD) of 0.05 mg mL^−1^ and a maximum adsorption capacity of 313.5 mg g^−1^. T4 bacteriophage-functionalized magnetic Janus MNRs have been used for the selective recognition and detection of E. coli (Figure 4D) [95]. MNRs were prepared by immobilizing T4 bacteriophage onto magnetic micromotors, which were propelled at speeds of up to 40 µm/s in complex biological samples, enabling “on-the-fly” capture of E. coli through specific bacteriophage–analyte interactions. A colorimetric readout based on AuNPs was used to detect the captured bacteria. The detection limit met the clinical cutoff for the rapid diagnosis of tract infections, and the platform demonstrated excellent selectivity for bacterial isolates from urine and serum samples from hospital patients. Table 1 lists a summary of colorimetric sensing assays using MNRs.

### 4.3. Fluorescence Sensing Using MNRs

**Figure 5:**
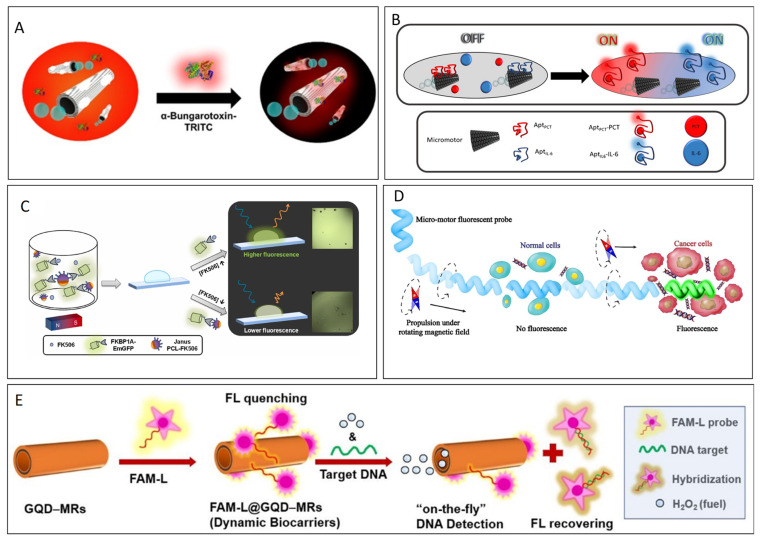
(**A**) Schematic of the detection of α-BTx-TRITC using MIP-MMs. Reproduced with permission from [98]. Copyright 2024, The Authors, Published by American Chemical Society. (**B**) OFF–ON MM-based dual aptassay for the simultaneous determination of PCT and IL-6. Reproduced with permission from [99]. Copyright 2024, The Author(s), Published by Springer Nature. (**C**) Schematic of the assay protocol for detecting FK506 using PCL/Fe_2_O_3_ magnetic-propelled micromotors. Reproduced with permission from [101]. Copyright 2023, The Authors, Published by Elsevier B.V. (**D**) Schematic showing the helical micromotors as fluorescent probes for DNA sensing. Reproduced with permission from [102]. Copyright 2022, Elsevier Ltd. (**E**) Self-propelled GQD–MRs as dynamic biocarriers for “on-the-fly” DNA detection. Reproduced with permission from [41]. Copyright 2023, The Authors, Published by American Chemical Society.

In recent years, MNR-based fluorescence biosensing platforms have garnered significant attention owing to their potential for the rapid, sensitive, and selective detection of various biological targets [10,16,21]. By integrating various functional materials and propulsion mechanisms, these systems offer enhanced capabilities for real-time, on-the-move biosensing in complex biological environments. In this section, we review the use of MNR-based fluorescence biosensing platforms to detect intracellular miRNAs, DNA, toxins, and biomarkers in clinical diagnostics and drug monitoring. The detection of multiple intracellular miRNAs is pivotal for cancer diagnosis and monitoring disease progression. A recent study demonstrated the preparation of an ultrasound-powered nanomotor-based fluorescent probe for the real-time detection of multiple miRNAs [96]. This approach uses gold nanowire (AuNW) nanomotors coated with graphene oxide (GO) and labeled with multicolored quantum dots (QDs). The fluorescence of the QDs is quenched by π–π interactions with the GO; however, upon binding to miRNAs, the distance between the QDs and GO increases, resulting in fluorescence “switching” from OFF to ON. This novel method offers significant advantages, including the rapid and highly sensitive detection of miR-10b and miR-21, enabling real-time discrimination between cancerous and normal cells. Furthermore, ultrasound-propelled nanomotors enhance probe–target interactions, improving the hybridization efficiency compared to passive methods. Li et al. devised a promising approach for H5N1 DNA detection using a DNA-stabilized silver nanocluster-based (Ag NC)-based platform [97]. The use of three-stranded DNA-stabilized Ag NCs allowed the fluorescence emission to darken when transformed into flexible single-stranded DNA upon interaction with the target molecule. This fluorescence-switching mechanism enables the highly sensitive and cost-effective detection of molecules such as H5N1 DNA, with a detection limit of 1.59 pM. This approach has the potential for wide applicability owing to its low cost and straightforward design. However, the requirement for precise DNA nanomachine manipulation introduces complexity. In the context of toxin detection, a new approach utilizing molecularly imprinted micromotors was developed for the rapid detection of α-bungarotoxin, a neurotoxin present in snake venom (Figure 5A) [98]. Micromotors were functionalized with specific recognition sites created by imprinting the target toxin onto the surface during fabrication. These micromotors, propelled by H_2_O_2_, demonstrated enhanced interaction with target analytes due to their autonomous movement, which increases the likelihood of target capture. The system demonstrated the ability to detect low concentrations of toxin in the range of 0.1 to 100 μg/mL in real-world samples, highlighting its potential applicability in clinical diagnostics. In neonatal care, rapid and accurate detection of sepsis biomarkers is critical because of the vulnerability of low-birthweight infants. A dual-aptamer-based micromotor biosensing platform was designed for the simultaneous detection of procalcitonin (PCT) and interleukin-6 (IL-6) biomarkers, two critical indicators of sepsis (Figure 5B) [99]. This system uses micromotors to rapidly mix the sample and interact with biomarkers, achieving detection within 15 min. This approach demonstrated an LOD as low as 0.003 ng/mL and a strong correlation with standard hospital methods. A novel approach for miRNA detection was developed using radio frequency plasma-modified magnetic Janus micromotors [100]. This method involves the functionalization of magnetic micro- and nanoparticles with hydroxyl groups, which were subsequently coated with Pt to enable autonomous movement via H_2_O_2_ propulsion. These Janus micromotors exhibit high speeds (up to 255 μm/s) and long operational lifetimes, making them ideal for biosensing applications. When applied to the detection of miRNA-21, changes in the fluorescence intensity and micromotor speed were used to indicate hybridization. This method shows potential for early cancer diagnosis, with promising results obtained using functionalized Fe_3_O_4_-OH/Pt micromotors. Monitoring immunosuppressive drugs such as tacrolimus (FK506) is essential in transplant patients owing to their high pharmacokinetic variability. A novel bioassay based on polycaprolactone-based magnetic Janus micromotors was developed to monitor FK506 via competitive binding to a fluorescent recombinant chimeric receptor (Figure 5C) [101]. This assay achieved high sensitivity with a detection limit of 0.8 ng/mL and a dynamic range of up to 90 ng/mL.

Magnetic micromotors have also been explored for detecting DNA released by apoptotic tumor cells, which is a cancer marker (Figure 5D) [102]. For instance, helical micromotors composed of polyvinyl alcohol (PVA), methacrylic acid (MA), and Fe_3_O_4_, which incorporated the fluorescent probe SYBR Green, have been devised for DNA sensing [102]. These micromotors demonstrated excellent navigation and movement in response to magnetic fields, and SYBR Green dye exhibited high sensitivity for detecting tumor DNA. This novel device that integrates micromotors with smartphone-based fluorescence detection offers a portable and accessible platform for real-time biosensing [103]. This system uses a 3D-printed holder to mount a smartphone equipped with an external optical lens and emission filters, thereby enabling the detection of fluorescence signals from micromotors modified with QDs. The micromotors were tested for mercury ion detection using ZnS@CdSe QDs and cholera toxin B detection using graphdiyne tubular catalytic micromotors. The smartphone-based system achieved a performance similar to that of a high-performance optical microscope, demonstrating its potential for field applications and point-of-care testing. The rapid and sensitive detection of bacteria is critical for quality control, environmental monitoring, and food safety. Traditional methods of bacterial detection, including culturing and biochemical assays, often require lengthy processing times and complex equipment. Recent advances in micromotor-based detection systems have offered promising solutions by integrating rapid mobility, selective recognition, and sensitive fluorescence-based readouts. For instance, researchers have used the “motion-capture-lighting” strategy employing Janus micromotors with a biphasic fiber rod structure for bacterial capture and fluorescence-based detection. In this study, flexible Janus fiber rods were fabricated by cryocutting aligned fibers produced by side-by-side electrospinning. The micromotors were functionalized with catalase on one side to generate oxygen bubbles for propulsion and with mannose on the opposite side for the specific recognition of FimH proteins found on the fimbriae of bacterial surfaces [104]. The propulsion mechanism of the micromotors was driven by oxygen bubbles generated by catalase activity. The bacterial capture process induced the fluorescence “lighting-up” of the micromotors due to the aggregation-induced emission effect of tetraphenylethene derivatives. Upon exposure to *E. coli* at concentrations of 10^2^ and 10^5^ CFU mL^−1^, the fluorescence of the Janus micromotors changed from blue to bluish-green and finally to green under UV light. The LOD was approximately 45 CFU mL^−1^, demonstrating the sensitivity of this approach. In another study, Janus micromotors, which are key indicators of food contamination, were developed to detect endotoxins released by enterobacteria [105]. These micromotors were fabricated using the Pickering emulsion approach, which enabled the simultaneous encapsulation of platinum nanoparticles for enhanced bubble propulsion and receptor-functionalized QDs for selective binding to the endotoxin target. Specifically, the micromotors targeted the 3-deoxy-D-manno-oct-2-ulosonic acid present in lipopolysaccharide molecules from Salmonella enterica. The detection mechanism was based on quenching of the native fluorescence of the QDs upon interaction with lipopolysaccharide endotoxins. Fluorescence quenching occurred in a concentration-dependent manner, allowing quantification of the endotoxin levels in the sample. The micromotor-based assay demonstrated remarkable sensitivity, with a detection limit as low as 0.07 ng mL^−1^ of endotoxin, significantly below the threshold considered toxic to humans (275 μg mL^−1^). Self-propelled microrobots composed of graphene quantum dots (GQD-MRs) have been used for sensitive DNA detection (Figure 5E) [41]. These GQD-MRs were biofunctionalized with a DNA biomarker and exhibited dynamic fluorescence changes upon hybridization with the target DNA. This “on-the-fly” detection system allowed for continuous monitoring of DNA concentrations within the nanomolar range. The GQD-MRs exhibited excellent sensitivity and selectivity, with detection limits as low as 0.05 nM. Table 2 lists a summary of the fluorescence sensing assays using MNRs.

## 5. Conclusions and Future Directions

Micromotor-based biosensing systems that use colorimetric and fluorescence approaches have emerged as powerful tools for the detection of bacteria, DNA, and other biomarkers, owing to their ability to navigate autonomously and detect targets in real time. The integration of autonomous propulsion, fluorescence switching, and target recognition into micromotors provides significant improvements over static methods, particularly for real-time and low-volume detection. However, there are several challenges to be addressed, including improving the specificity in complex biological environments, enhancing the portability of external control mechanisms, and ensuring the scalability of these systems for practical use in clinical diagnostics. Future studies should focus on expanding the range of detectable targets by exploring additional functional materials for micromotor modification. To enhance their utility, future MNR systems should aim to simultaneously detect a broader range of analytes. This can be achieved by incorporating multiplexed detection capabilities, enabling the recognition of multiple targets, such as different bacterial strains, toxins, or biomarkers, in a single assay. The integration of specific recognition elements, such as antibodies or aptamers, into micromotors could expand their scope of application in clinical diagnostics, food safety, and environmental monitoring. The propulsion mechanisms of MNRs, particularly enzyme-powered, bubble-propelled, and light-driven systems, can be further optimized to improve their efficiency, speed, and control. Enhancing the self-propulsion capabilities of MNRs in low-concentration environments as well as their maneuverability in complex biological or environmental matrices remains a key challenge. To realize the practical applicability of MNR-based biosensors, efforts should focus on developing portable and user-friendly detection platforms. The integration of smartphones, handheld readers, and point-of-care devices is critical for enabling rapid on-site detection without the need for specialized laboratory equipment. Innovations in 3D printing, microfluidics, and low-cost optical systems can drive this transition from the laboratory to practical applications. With continued advancements in materials science, propulsion mechanisms, and sensor integration, MNR-based biosensors have the potential to revolutionize diagnostic technologies and environmental monitoring applications.

## Figures and Tables

**Table 1:** Summary of colorimetric sensing assays using MNRs.

Material	Propulsion	Principle	Application	Reference
HRP/SDS	Marangoni effect	Enzyme-mediated colorimetric assay	Detection of H_2_O_2_ in water and clinical samples	[87]
PEDOT/Ni/Pt tubular micromotors with anti-cortisol antibodies	H_2_O_2_	Immunoassay-based colorimetric assay	Detection of cortisol	[88]
Single-wall carbon nanotube–Fe_2_O_3_/MnO_2_ micromotors	H_2_O_2_	Hydroxyl-radical-mediated colorimetric assay	Detection of phenylenediamine isomers	[89]
Ultra-small histidine-modified Fe_3_O_4_ nanoparticles	Enzyme/NIR light	Enzyme-mediated colorimetric assay	Detection of cholesterol	[65]
Fe_3_O_4_@SiO_2_/MNR-Ab1s	Magnetic field	Enzyme-linked immunosorbent colorimetric assay	Detection of procalcitonin in serum samples	[90]
Au@mSiO2@Pt Janus nanomotor	H_2_O_2_	Lateral-flow-immunoassay-based colorimetric assay	Detection of pepsinogen	[91]
MIL-88B@Fe_3_O_4_	Magnetic field	Hydroxyl-radical-mediated colorimetric assay	Detection of phenolic compounds	[92]
AuNR/PMO JNMs	NIR light	Lateral-flow-test-strip-based colorimetric assay	Detection of miRNA in serum samples	[93]
ZrO_2_/MnFe_2_O_4_/FeZr-MOF tubular magnetomotor	H_2_O_2_	Peroxidase-like colorimetric assay	Detection of glyphosate	[94]
Bacteriophage-functionalized magnetic Janus micromotors	Magnetic field	Bacteriophage-mediated colorimetric assay	Detection of *E. coli*	[95]

**Table 2:** Summary of fluorescence sensing assays using MNRs.

Material	Propulsion	Principle	Application	Reference
QD/ssDNA/GO/AuNW	Ultrasound	FRET-based fluorescence turn-on	Detection of miR-10b and miR-21	[96]
DNA nanomachine/Ag NCs	Self-propelled DNA	DNA-hybridization-based fluorescence turn-off	Detection of H5N1 DNA	[97]
Molecular Memory Micromotors (PEDOT/Ni/Pt NPs)	H_2_O_2_	Tetramethylrhodamine-based fluorescence turn-on	Detection of snake venom	[98]
GO/Ni/PtNPs	H_2_O_2_	Fluorescence off–on dual aptassay	Detection of procalcitonin	[99]
Fe_3_O_4_/Pt and Fe_3_O_4_-OH/Pt	H_2_O_2_	FAM-DNA-hybridization-based fluorescence turn-on	Detection of miRNA-21	[100]
PCL/Fe_2_O_3_	Magnetic field	Fluorescence competitive bioassay	Detection of tacrolimus (FK506)	[101]
PVA/MA/Fe_3_O_4_	Magnetic field	MNR-SYBR Green-based fluorescence turn-on	Detection of DNA	[102]
GQD–MRs	H_2_O_2_	FAM-DNA-hybridization-based fluorescence turn-on	Detection of DNA	[41]
ZnS@CdSe QDs	Magnetic field	ZnS@CdSe QD-probe-based fluorescence turn-on	Detection of cholera toxin B	[103]
PSMA-PEI-CAT-/PSMA-TPEC-Man-	H_2_O_2_	Aggregation-induced fluorescence turn-on	Detection of *E. coli*	[104]
Graphene QDs	H_2_O_2_	Fluorescence turn-off	Detection of endotoxin from *Salmonella enterica*	[105]

## Data Availability

No data were used for the research described in the article.
